# Assessment of a prototype for the Systemization of Nursing Care on a mobile device^1^

**DOI:** 10.1590/1518-8345.0898.2714

**Published:** 2016-07-04

**Authors:** Laura Cristhiane Mendonça Rezende, Sérgio Ribeiro dos Santos, Ana Lúcia Medeiros

**Affiliations:** 2 Professor, Faculdade Maurício de Nassau, João Pessoa, PB, Brazil.; 3 PhD, Associate Professor, Departamento de Enfermagem Clínica, Centro de Ciências da Saúde, Universidade Federal da Paraíba, João Pessoa, PB, Brazil.; 4 Doctoral Student, Centro de Ciências da Saúde, Universidade Federal da Paraíba, João Pessoa, PB, Brazil. Professor, Faculdade Internacional da Paraíba, João Pessoa, PB, Brazil.

**Keywords:** Informatics in Nursing, Nursing Care, Electronic Health Records

## Abstract

**Objectives:**

assess a prototype for use on mobile devices that permits registering data for the Systemization of Nursing Care at a Neonatal Intensive Care Unit.

**Method:**

an exploratory and descriptive study was undertaken, characterized as an applied methodological research, developed at a teaching hospital.

**Results:**

the mobile technology the nurses at the Neonatal Intensive Care Unit use was positive, although some reported they faced difficulties to manage it, while others with experience in using mobile devices did not face problems to use it. The application has the functions needed for the Systematization of Nursing Care at the unit, but changes were suggested in the interface of the screens, some data collection terms and parameters the application offers. The main contributions of the software were: agility in the development and documentation of the systemization, freedom to move, standardization of infant assessment, optimization of time to develop bureaucratic activities, possibilities to recover information and reduction of physical space the registers occupy.

**Conclusion:**

prototype software for the Systemization of Nursing Care with mobile technology permits flexibility for the nurses to register their activities, as the data can be collected at the bedside.

## Introduction

Information Technology (IT) has become part of people's daily life all over the world. The application and the use of technology and computer-based technologies for health care are an ongoing process^1^.

This accelerated development of scientific and technological modernization has produced new forms of knowledge construction and establishing relations with the labor world. It is believed that, in upcoming years, the advances of computer technology will revolutionize the processes at all levels of nursing work at health institutions and offer operational and strategic benefits for professional organization and practice^2^.

It is important to clarify that, often, the term health technology is associated with the machinery developed for individual rehabilitation and survival. It should be highlighted that this concept can be expressed in different ways: hard technology, which refers exactly to the common-sense idea of machines, organizational standards and structure; soft-hard technology, represented by the theoretical knowledge that will support the understanding of the health work process and soft technology, evidenced by the interpersonal relations aimed at attending to the user's needs^3^ ^-^ ^4^. In this study, the goal is to evidence the contribution of both hard and soft-hard technology in the condition of nursing work.

As a result of the evolution in these technologies and the constant miniaturization process of computers, today, large amounts of information can be obtained and carried digitally using portable devices, such as handhelds, smartphones and tablets^5^.

Studies verify that the nurses' difficulty to employ other computer tools than mobile devices is to transport the information collected from the patient to the microcomputer. As a result of the distance between the location of the hardware and the patient's bedside, the nurse registered the data collected from a patient on paper and later transcribed it. That is one of the main problems in using fixed computers to register nursing practice, as the care activity involves the professionals' mobility to attend to different inpatients^6^.

In the context of nursing care at the Neonatal Intensive Care Unit (NICU) of the teaching hospital where this study was undertaken, the Nursing Care Systemization (NCS) was not developed. The records were handwritten and the clinical evolution was not standardized, occupying considerable physical space and, in addition, demanding the nurse's time to make notes.

Thus, mobile computing emerges as an innovative technology for nursing care, through its application via a mobile device to other computers, via an interface of integrated and planned wireless network interface. The parallel use of mobile computing and the access to this kind of network can undoubtedly help considerable in the health professionals' daily life^7^.

With the mobile device at hand, information on the patient can be accessed, collected and documented at the bedside, steps of the Nursing Process can be developed and the professionals' need for mobility in patient care actions can be monitored. The time spent to document the activities can also be reduced and the probability of losing information can be decreased. This starts to be stored on the device instead of paper, which demonstrated how the characteristics of flexibility and dynamism converge mutually and contribute to the productivity of nursing care^8^.

In that perspective, this study was aimed at developed an assessing a prototype for a mobile device, which permits registering data for the Systemization of Nursing Care at a Neonatal Intensive Care Unit.

## Method

An exploratory and descriptive study was undertaken, also characterized as an applied methodological research, developed at a teaching hospital located in the city of João Pessoa, Paraíba, between March and October 2014.

The development of the prototype followed three phases: 1^st^ phase - definition, in which the information for processing will be presented, the function, the performance of the program, the restrictions and the interfaces; 2^nd^ phase - development, when the data entry, the project architecture, the procedure details for the implementation and translation to the program language and tests on the applicability of the prototype are structured; and the 3^rd^ phase - maintenance, characterized by the correction of errors and adaptations to the users' requirements (nurses).

In the elaboration of the software prototype, a database was used which the nurses at the unit had constructed and validated. The main technological tools used to develop the software were: the program language Ruby, Ruby on Rails and JavaScript; the Bootstrap framework; the production server Ubuntu Linux, Nginx Webserver and the Database Management System*.*

Based on the implementation of the software at the Neonatal ICU, the nurses participated by using it in practice, and then by assessing the prototype. On this occasion, five professionals affiliated with the teaching hospital participated, who worked at the Neonatal ICU and were present in September and October 2014, when the system was maintained.

Concerning the participants' characteristics, the length of education ranged between 10 and 30 years and all of them held some kind of specialization degree - in education, collective health, occupational health and pediatric nursing; only one nurse held a Master's degree in Nursing. The length of professional experience at the unit ranged between 10 and 12 years. They were also asked about knowledge on informatics and they unanimously affirmed that they had never taken a course or training in information technology.

For the participants to maintain the system, a tablet was used, 7", dual core, Android 4.0, connected to the unit's wi-fi. It is important to highlight that the system ideally functions on any mobile device (smartphone or tables) with Internet access, without the need for minimal configurations. The software can also be used on computers, as its development permits the use on different platforms.

To assess the suitability of the protocol for mobile devices to the reality of the teaching hospital's Neonatal ICU, the participants were interviewed to get to know their opinion on the difficulties to handle the system, the importance of the prototype for NCS and the suggestions to improve it. The data were analyzed through a qualitative approach and Bardin's content analysis was chosen for the analysis.

Concerning the ethical aspects, the orientations inherent in the research protocol in National Health Council Resolution 466/12^9^ were followed. The project was forwarded to the Research Ethics Committee, approved and registered in the National Information System on Ethics in Research involving Human Beings (SISNEP), under CAAE-25890914.5.0000.5183, on March 13^th^ 2014.

## Results

The results appointed the two research phases: the first that showed how the prototype was developed and the second that assessed the prototype on a mobile device.

### Phase 1 - Development of the Prototype for Nursing Care Systemization

The prototype was developed through the use of a database the nurses from the unit had validated, which presents the following empirical data, used in care practice: identification of the infant, anthropometric data, vital signs and motive for the hospitalization. The data on the assessment parameters of the infant's health condition and which supported the construction of the care plan were elaborated in view of the following human needs: shelter, thermal regulation, oxygenation, hydration, nutrition, cutaneous-mucous, physical and corporal integrity, exercise, physical motility, sleep and rest, perception, endocrine regulation, need for elimination, therapeutics, communication and, finally, the nurse's supplementary notes. What the elaboration of the care plan is concerned, 273 assertions are presented, 143 of which related to the Nursing Diagnoses and 130 to the Nursing Interventions, constructed based on the ICNP 1.0. 

**Figure 1:**
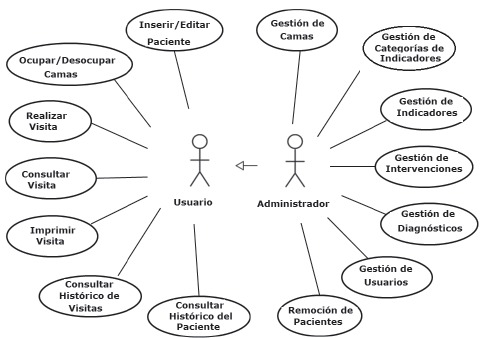
Diagram of case functions according to type of system user

Concerning the system functions, there are two types of credentials: standard users, which in this study refer to the nurses from the Neonatal ICU, and the administrator, in this case the researcher. The standard user can do the following: include and edit patients, occupy/void beds, visit the patients, consults the data on a visit done, print data, consult the time of the visit and the patient. Besides the above actions, the administrator can also: manage beds, include categories of indicators, which refer to the human needs, include nursing diagnoses and interventions, manage users, besides excluding patient information, as can be observed in the diagram of usage cases, shown in Figure 1.

**Figure 2:**
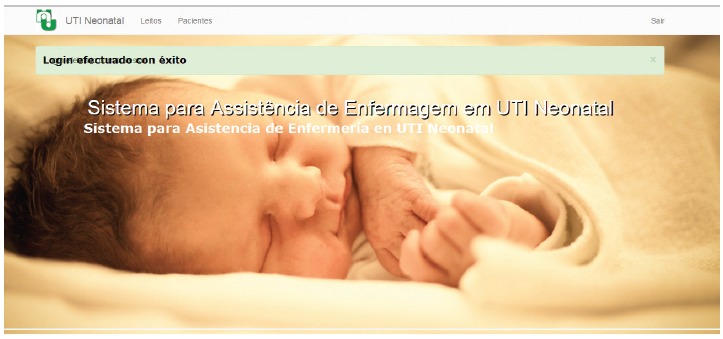
Initial screen of the system after the login

The use of the Nursing Care System at the Neonatal ICU depends on the access to the link: www.utineonatal.bitmine.com.br. The initial screen of the software is displayed in Figure 2. To get access, the user name or e-mail and password need to be completed. Both are registered by the system administrator. The screen subsequent to the access (Figure 2) shows a message confirming that the login was successful. Thus, as a standard user, the nurse can access the software through the following options: 'beds', 'patient' or 'exit' the system.

**Figure 3:**
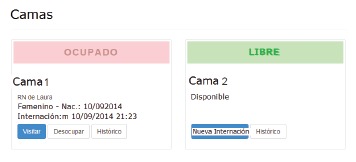
Images shown to inform on available and occupied beds

When selecting the option 'beds', the nurse sees the number of beds available at the unit. Occupied beds can be identified rapidly (pink), as well as the beds available for a new hospitalization (green), as shown in Figure 3.

For the free beds, a new hospitalization can be registered or the history can be accessed, indicating the data and visits to the infants who were occupying that bed earlier. To start an admission, the nurse chooses the free bed and selects the option 'new hospitalization'.

Next, the nurse needs to follow the admission process or choose to research the patient as, in case of earlier hospitalization at the unit or in another bed, the patient's identification data can be retrieved.

The requested data, requisites to proceed with the patient admission, are: name of the infant, patient history number, date and time of birth, sex, blood type, one and five-minute Apgar score, type of delivery and gestational age, name and sex, besides the admission date and time, which have to be modified because, if they are not, the date and time they are being used in the system continue, the reason for hospitalization, that is, why the infant was admitted to the unit, the origin and transportation conditions, besides data on the physical examination. After including the data on the physical examination, the development of the care plan starts.

**Figure 4:**
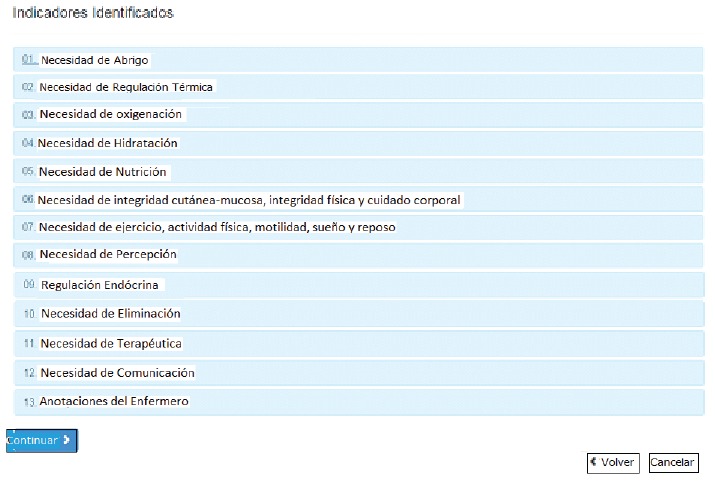
System categories for the development of NCS for infants

Each need (Figure 4) comes with its respective infant assessment items, regarding the "needs for thermal regulation" for example, the nurse can indicate if the infant is hypothermic, hyperthermic or normothermic. Under "oxygenation needs", the following assessment items are available: respiratory changes, aspiration of secretions, respiratory auscultation, respiratory frequency, supplementary oxygenation, types of breathing, retraction, cough and other considerations. 

**Figure 5:**
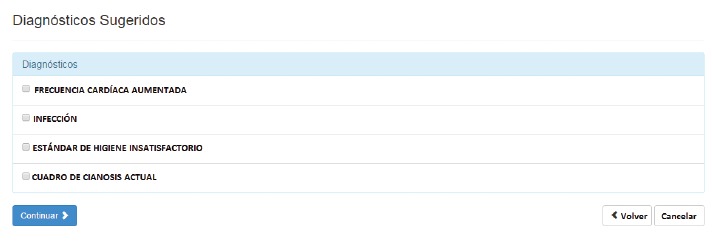
Suggested Nursing Diagnoses

After completing the needs and their respective evaluation items, the system suggests a list of Nursing Diagnoses, associated with the information made available earlier, to be selected by the nurse who will use the decision model based on clinical reasoning to define the diagnoses relevant to the condition of the infant being assessed (Figure 5).

After selecting the Nursing Diagnoses, the next step is the elaboration of the plan of associated interventions, according to the logic of the principles shown earlier.

After concluding the selection of the Nursing Interventions, the nurses terminates and saves the nursing care records, which are filed in the system. All information produced can be printed (producing a .pdf file), when finishing the inclusion of information or at any other time. 

Concerning the system functions for the user/administrator, the initial screen is similar to the screen displayed to standard users. The main difference relates to the other functions the system offers for this kind of user. The administrator can make changes in the system database, that is, new information can be included in the functions considering the Nursing Process: needs, assessment items, nursing diagnoses and interventions. The same person is responsible for managing the users, that is, for registering the nurses who can use the system.

### 2**^nd^** Phase - Assessment of the prototype for Nursing Care Systemization on mobile devices

To assess the applicability of the system developed for mobile technology in care practice, after the software maintenance period, interviews were held with five nurses.

Through the assessment of the prototype, three categories could be identified: appointing system maintenance difficulties, acknowledging the importance of the prototype for NCS on mobile devices and suggesting changes to adapt to the care reality. 

### Category 1 - Appointing system maintenance difficulties

*[...] the main difficulty is that I'm not good with informatics (laughs), but... I was able to handle it, I was able to open it, do the evolutions (E1); [...] in my case, I'm a starter, in systemization as well as informatics [...] I experience some difficulty (E2); [...] no, difficulty really, I couldn't find anything [...] I was able to advance through all steps. I found it was easy, I've already got this small one, so... (referred to the mobile phone)) (E3); [...] no, no difficulty. I have the same table, so I had no problem (E5).*

The reports demonstrate that some nurses experienced difficulties to use the tablet, which are related to the professionals' lack of experience with the technology and the fact that they had never used this kind of mobile technology in their work process, and mainly because it is a touchscreen device*.*

On the opposite, some nurses did not experience difficulties to handle the mobile technology, affirming that they have a device similar to the table, either a mobile device like a smartphone or technology identical to the type used in the study. 

### Category 2 - Acknowledging the importance of the prototype for NCS on mobile devices 

*[...] it (tablet) grants you freedom to move and do the physical examination wherever you are, you already evolve and proceed with your work process (E1); [...] with the mobile device, I can go somewhere else if the computer is occupied, I can do it standing, you're very limited on the computer [...] I think that, in general, it gets more organized, the nurse, whoever it is, will follow those steps, will standardize (E3); [...] I gain time, I manage my actions better (E4); [...] the fact that the device is mobile grants the nurse mobility [...] agility to elaborate the care plan, a safe register, a file without occupying physical space, besides saving time in the implementation of the NCS (E5).*

The statements highlighted show that there is a consensus among the nurses on the importance of an application for NCS using mobile devices. The advantages mentioned mainly refer to requisites like mobility and agility for the patient evolution and elaboration of the care plan, thus optimizing the time, besides the flexibility granted in the management of care actions when using the tablet.

### Category 3 - Suggesting modifications in the system to adapt to the care reality

*[...] for us to visualize the end better, it would need more colors [...] the answers should be more colored (E1); [...] to adapt to the infant, some things need improvements, the part about the child's assessment (E4); [...] although I liked it, I would include the option to save the data as they are informed to the system [...] condense the information further, reducing the volume of paper needed for printing (E5).*

These reports demonstrate the need for changes in the final step, in which all information included and the selected items are listed. This screen produces the document that is printed and attached to the patient history. The nurses also suggested changing the color between questions and answers for any professional to see the information easily. In addition, the need was highlighted to automatically save the information and to adapt some software items to the particularities of infant assessment in critical conditions. Other suggestions referred to the possibility to compact the final information to reduce the size of the PDF document and, consequently, to reduce the consumption of printing paper. 

## Discussion

It cannot be denied that the technological advances have increasingly influenced the health care practices. In that context, the technology has also greatly influenced the nurses' daily work. In recent years, the use of IT, including computers, portable digital devices and the Internet, has advanced in nursing knowledge, permitting the construction of a link between the art and science of nursing. In all spheres of these professionals' practices, in nursing research and in the insertion of informatics into nursing education, the technological resources play a very important role. If used correctly, technology is a way to save time, helping to offer high-quality nursing care, besides contributing to the nurses' proficiency^10^.

Nevertheless, the nursing professionals' lack of proximity with the computerization process is still present nowadays. In this study, it was verified that none of the nurses had participated in a computer training course, which could also result in the difficulty to adapt the system for mobile devices to their daily work, despite their daily use of smartphones. 

With a view to minimizing the shortages in technology use in care practice, interventions can also take place during undergraduate nursing programs. In the same perspective, a pilot study undertaken at the University of Philadelphia in the United States sought strategies to include the table into the daily reality of nursing undergraduates and obtained similar results. Initially, the difficulties and resistance existed but, as a result of the use, the users gradually adapted to the particularities of the mobile devices^11^.

Since 2003, in New York City, nurses from a health service who engage in home visits use tablets to document patient information. The mobile devices helped to computerize and handle different forms used during the visits^12^. This means that the inclusion of technologies into the nurses' daily work grows daily and that the professionals need to get familiar with these advances to adapt to the new reality.

Authors affirm that the mobile devices offer great advantages, including the fact of being portable (capable of being transported relatively easily), usable and functional, easy to connect and communicate with the users and with other devices. Another important aspect is the user's facility to move, as the mobile device fits into the hand palm, improves the visual quality and is more comfortable, light, easy to use and discrete^13^.

In a study on the use of tablets to register clinical information involving North American nurses, the authors concluded that these mobile devices are convenient. In one of the reports, the participants highlighted that the nurses are always short of time and interested in anything that can simplify their lives and grant them some more free time^14^.

Concerning the interface, some authors affirm that, when assessing software based on the viewpoint of the end user, one of the most important factors is the communication interface between the user and the system, which should be easy to learn and intuitive because, to reach an objective, the user should follow "certain steps" easily. In this study, the nurses reported good acceptance of the program interface, merely suggesting some color changes, suggestions observed in earlier studies, where nurses highlighted that the software assessed should have more contrasting colors^15^.

It was also observed in this study that some aspects of the system that permit assessing the infant's health condition, like the assessment of reflexes for example, need to offer judgment items that reflect the particularity of these clients. In this respect, the authors emphasize that the system developers have faced a permanent challenge to enhance the activity flow, reduce the professionals' work burden and adapt the design and content of the technological devices and systems to the reality of the nurses' care practice^16^.

Authors emphasize that, besides the contributions to care practice, the technological advances grant the nurses the opportunity to direct their professional destiny, adapting technological resources to care, helping them to envisage emerging trends in health as challenges and opportunities to grow in the career. New tools are available, new areas and new work, demanding experts in any country, a vast number of opportunities available to who decides to incorporate technological information in daily practice^17^.

In this study, the nurses' comments and suggestions permitted identifying the difficulties, importance and strategies to better adapt the prototype to the care reality of the Neonatal ICU, besides the advantages the system can offer in daily nursing work. 

## Conclusion

In this study, a system was developed that allowed the nurses to systemize nursing care at Neonatal ICU through the use of a tablet. When included in the care reality to support the practice, even if as a test, the research revealed that the nurses experienced difficulties to use the mobile devices, but that the advantages surpassed these obstacles.

It was verified that an NCS system through mobile technology enhanced the flexibility of the nursing records, because the data were collected at the bedside and the Nursing Process was dev eloped anywhere at the service, as the technology does not depend on wires to function. It is also highlighted that, through the use of a database compatible with nursing practice at the Neonatal ICU, all steps of the Nursing Process could be developed and the gap between theory and practice could be reduced. 

Another advantage observed was the optimization of the nurses' time, as the computerization of bureaucratic activities resulted in greater efficacy and efficiency in the nursing records, permitting time savings that can be reverted to client care and the standardization of the infants' evolutions, essential for the continuation of client care and assessments by other professionals.

The results of the nurses' assessment of the system showed that the difficulties were mainly related to the professionals' lack of familiarity with the technology, more specifically with the touchscreen. Even in cases of usage difficulties, however, causing some aversion to include a new information registering method, the nurses, through frequent use of the software and the mobile device, adapted well to the innovation.

This study considers a prototype, further changes are due to better adapt the database to the reality of infants in intensive care, as well as changes in the system functions and structure to guarantee its perfect functioning. Despite this need for changes, the system was well adapted to the reality of the service where the study was developed and as an excellent product to be implemented at the unit without further difficulty, considering that the institution simply needs to offer a wireless internet router and a mobile device (tablet)*.*

The information technologies associated with the mobile devices clearly contribute to the nurses' work process, demanding further investments in studies aimed at equipping the Nursing Process and inserting new technologies into these professionals' daily work, not only at Neonatal ICU, but at all nursing services and workplaces.
